# From an Enteroatmospheric to an Enterocutaneous Fistula Using a Condom

**DOI:** 10.7759/cureus.74209

**Published:** 2024-11-22

**Authors:** Sofia Gaspar Reis, Patrícia Bernardo, Nuno Mendonça, Hélder Além, Zara Caetano

**Affiliations:** 1 General Surgery, Centro Hospitalar Barreiro Montijo, Barreiro, PRT

**Keywords:** condom technique, enteroatmospheric fistula, enterocutaneous fistula, negative pressure wound therapy, open abdomen

## Abstract

An enteroatmospheric fistula (EAF) is one of the most feared complications of damage control laparotomy. Its management is highly challenging, often requiring multiple surgeries and prolonged hospitalization. It is a serious condition, and despite significant improvements in mortality rates due to advancements in intensive care, the rate remains substantial. We describe the case of a 75-year-old male who presented to the emergency department with abdominal pain one day after being discharged from another hospital following an elective converted cholecystectomy. He underwent emergency median relaparotomy, revealing fecal peritonitis and jejunum leakage. Following the jejunal segmental resection with mechanical anastomosis, we chose to leave the abdomen open. Eight days later, an EAF was established, and the abdomen was classified as grade 4 according to Bjork (classification of 2016). To manage this complication a four-step technique was employed: latex condom-EAF anastomosis, fistula ring creation, negative pressure wound therapy (NPWT), and adaptation of an ostomy bag. Nine weeks later, the wound was fully healed, and the stoma completely matured. Several recent reports have discussed the treatment of this condition. Techniques employing a baby bottle nipple, silicon plug, and floating stoma have shown promising results. NPWT was considered to increase the risk of fistula formation for many years, but additional studies have demonstrated its safety. No gold standard therapy has been established for EAF treatment; therefore, decisions rely on the surgical staff's experience. This technique for effluent control in patients with a Björk grade 4 abdomen and established EAF is easily reproducible and safe.

## Introduction

An enteroatmospheric fistula (EAF) represents a catastrophic complication that often occurs in the setting of an open abdomen (OA) [1].

"True fistulas" refer to abnormal connections or passages that form between two epithelial-lined surfaces, such as between two organs or between an organ and the skin, and typically have a well-defined tract with epithelialization. Unlike true fistulas, an EAF is characterized by abnormal communication between the gastrointestinal tract and the external atmosphere. The absence of a defined tract and the presence of poorly vascularized surrounding tissue make spontaneous healing nearly impossible [2].

In the past decades, mortality rates were as high as 70%. However, with advancements in modern invasive care and improved surgical techniques, the current mortality rate has decreased to approximately 40% [3]. In addition, this pathology increases the hospital length of stay and its inherent costs by at least fourfold [4].

The OA technique, first described by McGosh in 1897, is a key component of damage control surgery [5], allowing for rapid stabilization of critically ill patients. It is utilized in emergencies, such as managing intra-abdominal hemorrhage and treating intra-abdominal hypertension and sepsis, while also providing easier access to the abdominal cavities for further interventions [6].

Although OA offers significant benefits, it also introduces risks, such as hollow viscera perforation, particularly due to necessary manipulations like abdominal dressing changes. These interventions can be traumatic to the already swollen and fragile bowel, potentially leading to the development of an EAF with an incidence rate from 14% to 25% [7,8]. As stated by Björck et al. [9] on the OA classification, EAFs typically arise in Grades 3 and 4 OAs.

The classification of EAFs considers their anatomical location (proximal or distal), fistula output (low: <200 mL, moderate: 200-500 mL, high: >500 mL), and their position within the OA (deep or superficial) [7].

Despite advances in surgical techniques and critical care, managing EAFs remains a significant challenge and requires a multidisciplinary approach. A widely recognized acronym is "SNAP," which stands for Sepsis and Skincare, Nutritional support, identifying the intestinal Anatomy, and devising a surgical Procedure to address the fistula. Major challenges in managing this condition include protecting the exposed bowel and controlling fistula effluent [10].

This case report aims to describe the application of the four-step technique for effluent control in patients with EAFs.

## Case presentation

We present the case of a 75-year-old male admitted to the emergency department with abdominal pain, nausea, vomiting, and sweating. He had undergone a converted cholecystectomy (midline laparotomy) at another hospital four days earlier and was discharged the day before. According to his medical history, he had hypertension. There were no other relevant health records.

On the physical examination, the patient had a distended, tympanitic abdomen that was tender to palpation across all quadrants, with guarding and signs of peritoneal irritation. There was also a hematoma at the site of a recent midline supra- and infraumbilical laparotomy wound.

**Figure 1:**
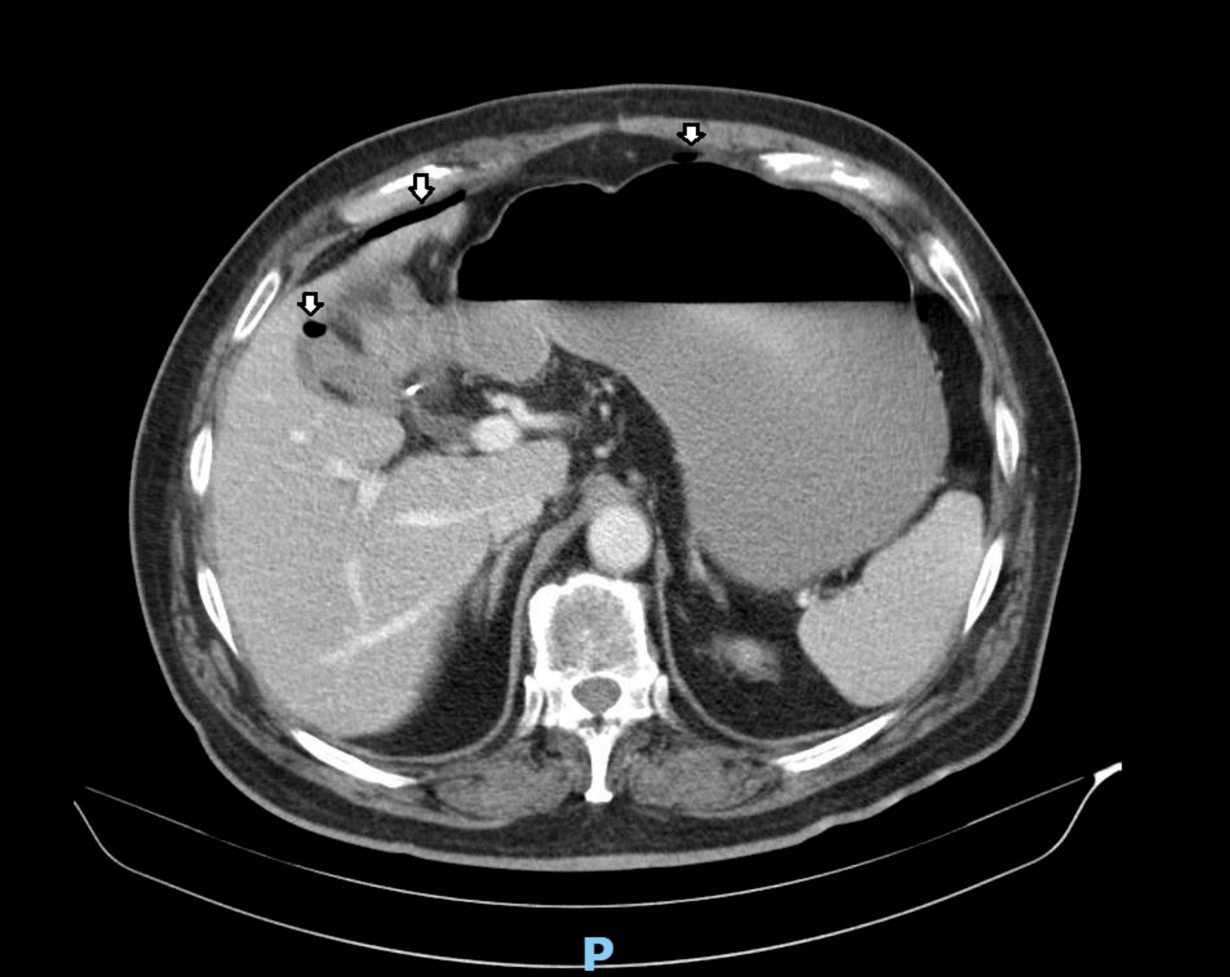
CT scan revealing pneumoperitoneum

**Figure 2:**
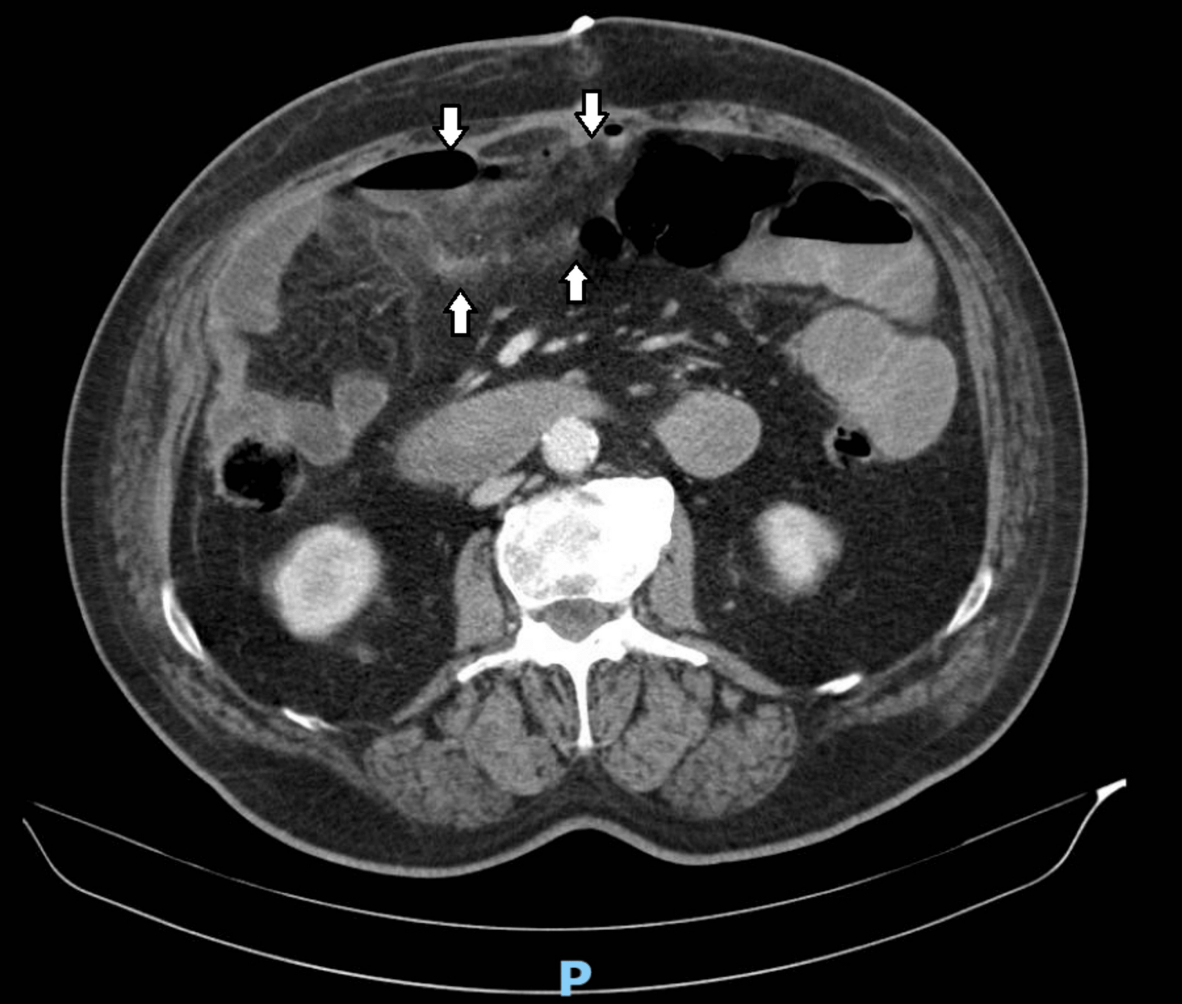
CT scan image revealing fat stranding and a small volume of loculated fluid suggesting a collection approximately 30 mm in diameter

Laboratory tests indicated hypokalemia and elevated inflammatory markers. A contrast-enhanced abdominal and pelvic CT scan revealed pneumoperitoneum with several gas bubbles near the anterior abdominal wall and within the interloop area, located at the transition between the right iliac fossa and right flank, adjacent to a loop of the small intestine. This was associated with significant fat stranding and a small volume of loculated fluid, suggesting a small collection approximately 30 mm in diameter. Free intraperitoneal fluid was also noted in the subhepatic area and right iliac fossa. These findings suggested a probable intestinal perforation (Figure 1, Figure 2). 

The patient underwent emergent exploratory relaparotomy, during which hemoperitoneum was identified, but no clear source of hemorrhage was found. In addition, fecal peritonitis with necrotic areas of the small intestine and two jejunal perforations were identified. A segmental enterectomy was performed with latero-lateral mechanical anastomosis (gastrointestinal anastomosis stapler (GIA) 80 mm, blue cartridge), and a laparostomy was created using negative pressure wound therapy (NPWT) to allow for a second-look procedure. The patient was subsequently admitted to the intensive care unit (ICU) due to septic shock.

During his ICU stay, the patient required invasive ventilatory support and vasopressor therapy. On the second postoperative day, the laparostomy was reviewed, and since there was no evidence of contamination in the abdominal cavity, it was closed, and a passive drain was left near the jejunal anastomosis.

**Figure 3:**
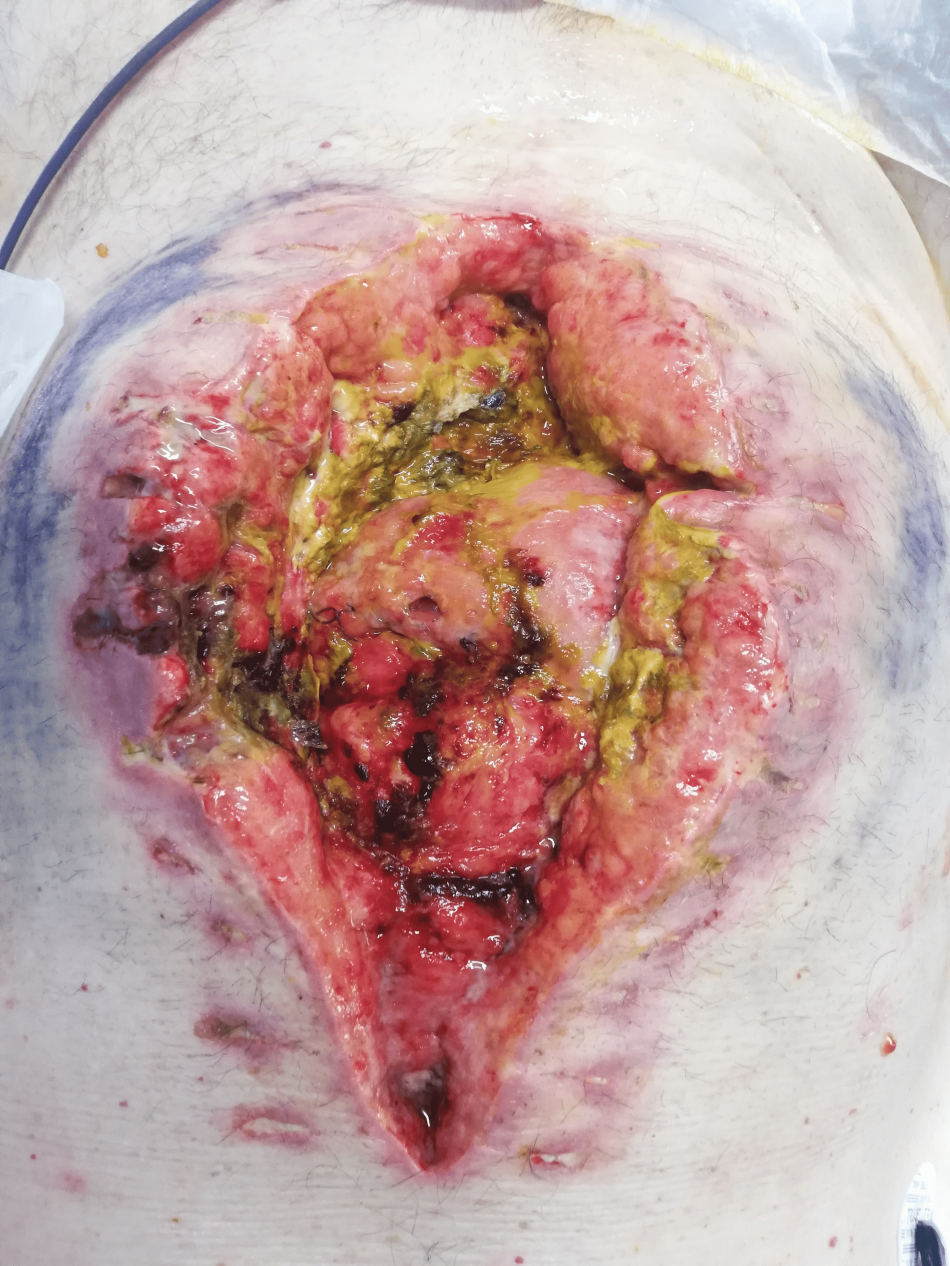
Frozen abdomen and anastomotic leak

On the eighth postoperative day, due to the drainage of enteric content through the abdominal wound (unquantified drainage), another surgical intervention was planned to address the complication. Intraoperatively, it was found that the abdominal cavity could not be accessed due to a frozen abdomen (Grade 4 on the 2016 Björck classification) and an anastomotic leak of about 3 mm was found (Figure 3).

The NPWT was applied with isolation of the EAF using a nipple and leveling paste.

It was noted that the nipple technique had poor results, with leakage of the fistula’s drainage content into the negative pressure dressing. It was believed that the pressure was being transmitted to the small intestine, potentially contributing to increased drainage volume and enlargement of the fistula opening.

Two days later, in the operating room, we employed a four-step technique: latex condom-EAF anastomosis, creation of a fistula ring, application of NPWT, and adaptation of an ostomy bag, as described below.

Technique

Step 1: Condom-EAF Anastomosis

**Figure 4:**
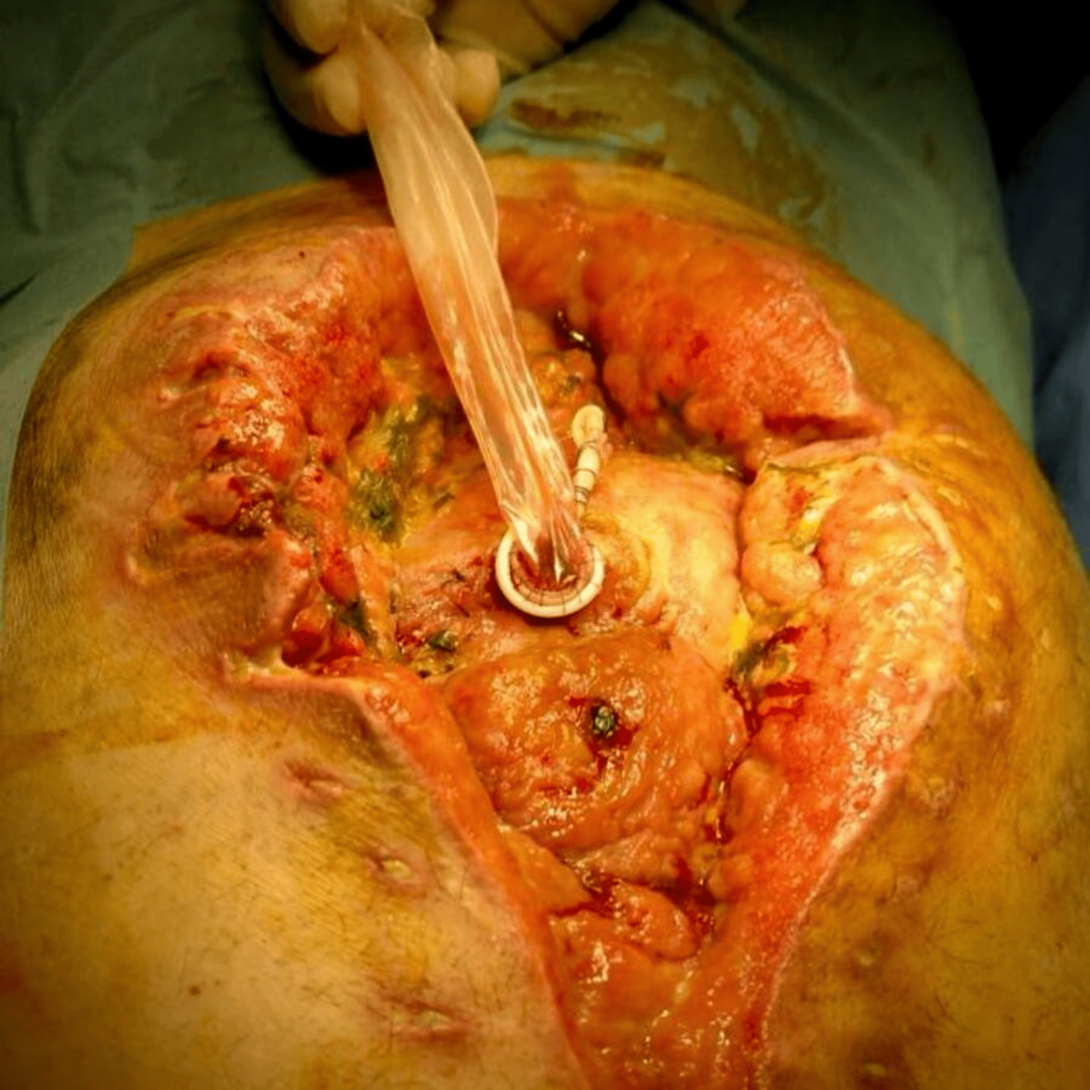
Condom-fistula anastomosis

**Figure 5:**
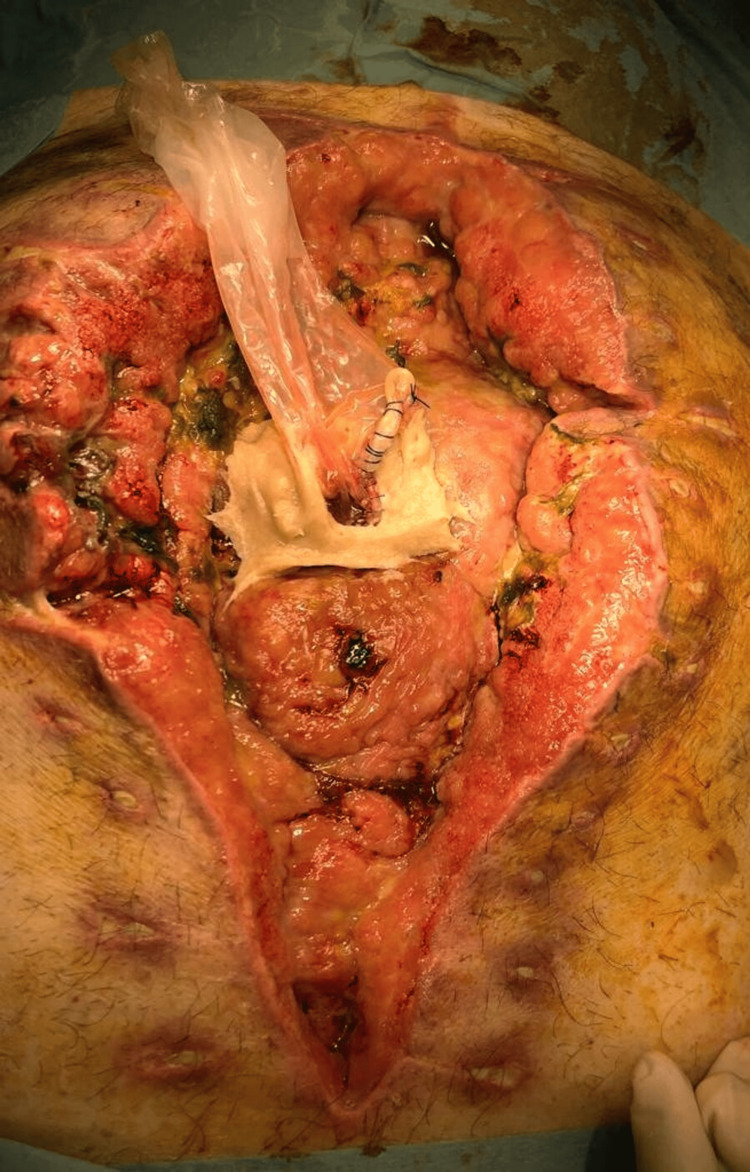
Condom-fistula anastomosis with leveling paste to minimize leakage

A latex rubber condom is sutured with continuous 4-0 nonabsorbable polypropylene to the EAF opening with full-thickness bites. The ending part of the condom is cut. After completing the anastomosis, an insulating barrier is created using a leveling paste to minimize leakage. A leak test is then performed to determine if additional suture reinforcement is needed (Figure 4, Figure 5). 

Step 2: Protective Ring Creation

**Figure 6:**
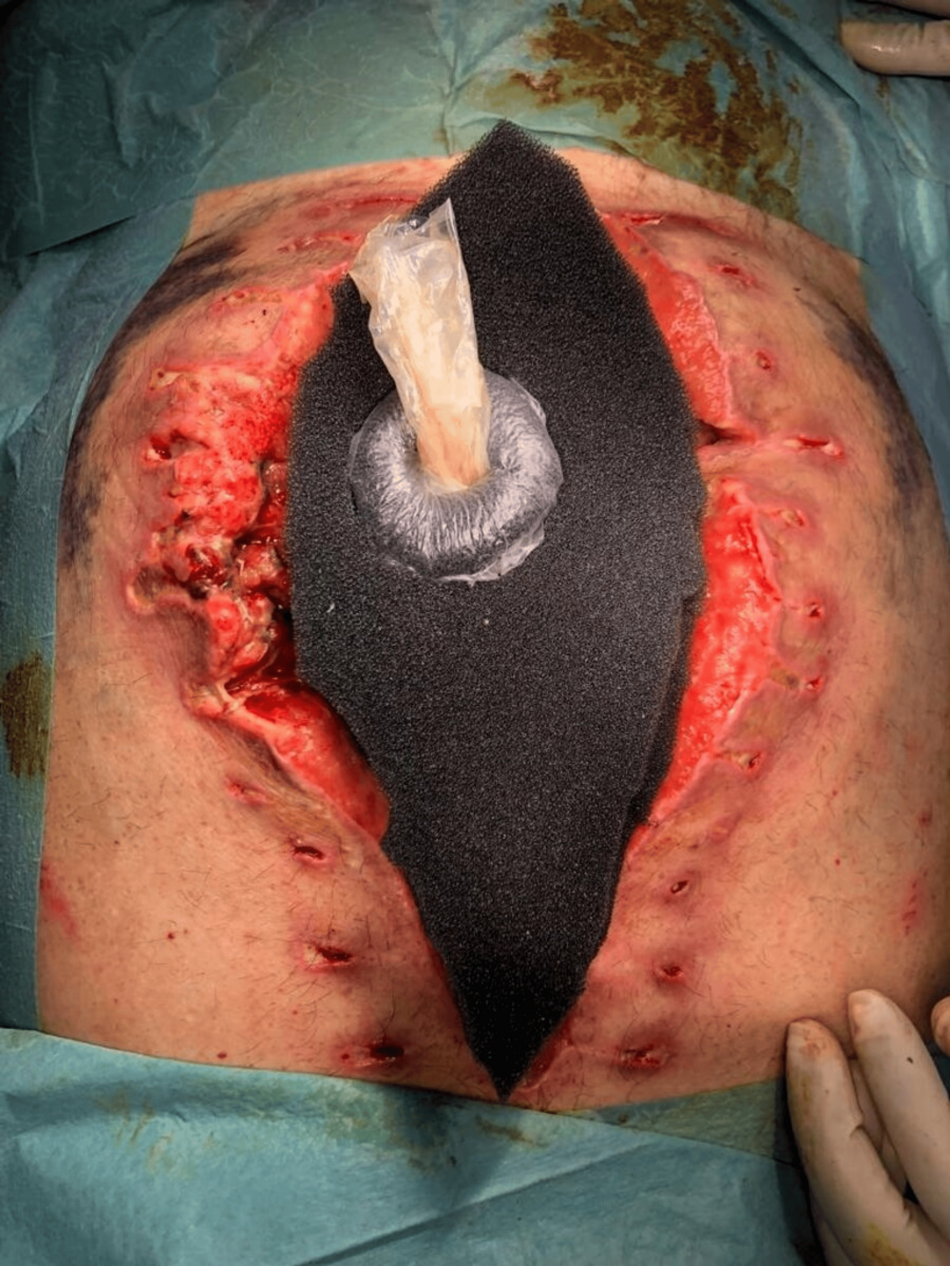
Protective ring and negative pressure wound therapy application

A protective ring is created using the foam from the NPWT dressing itself. This ring is then sealed with an occlusive drape to prevent the transmission of negative pressure between the foam layers. Although this ring can be made with other materials, we chose to use the foam from the NPWT dressing to reduce costs. The ring is then positioned around EAF-condom anastomosis (Figure 6). 

Step 3: NPWT Therapy

The black foam is then cut to fit the shape of the remaining abdominal wound, leaving about 0.5 cm from the wound edges. An occlusive drape is applied over the entire abdomen to cover the foam completely. Next, the drape is cut along the inner contour of the ring so that the condom is not covered by the adhesive. A small hole (approximately 1 cm in diameter, varying by the NPWT brand) is created in the drape, positioned away from the location of the condom. The NPWT suction device is then adapted above this hole and connected to the machine, with the pressure set between -125 and -100 mmHg (Figure 6).

Step 4: Ostomy Bag

**Figure 7:**
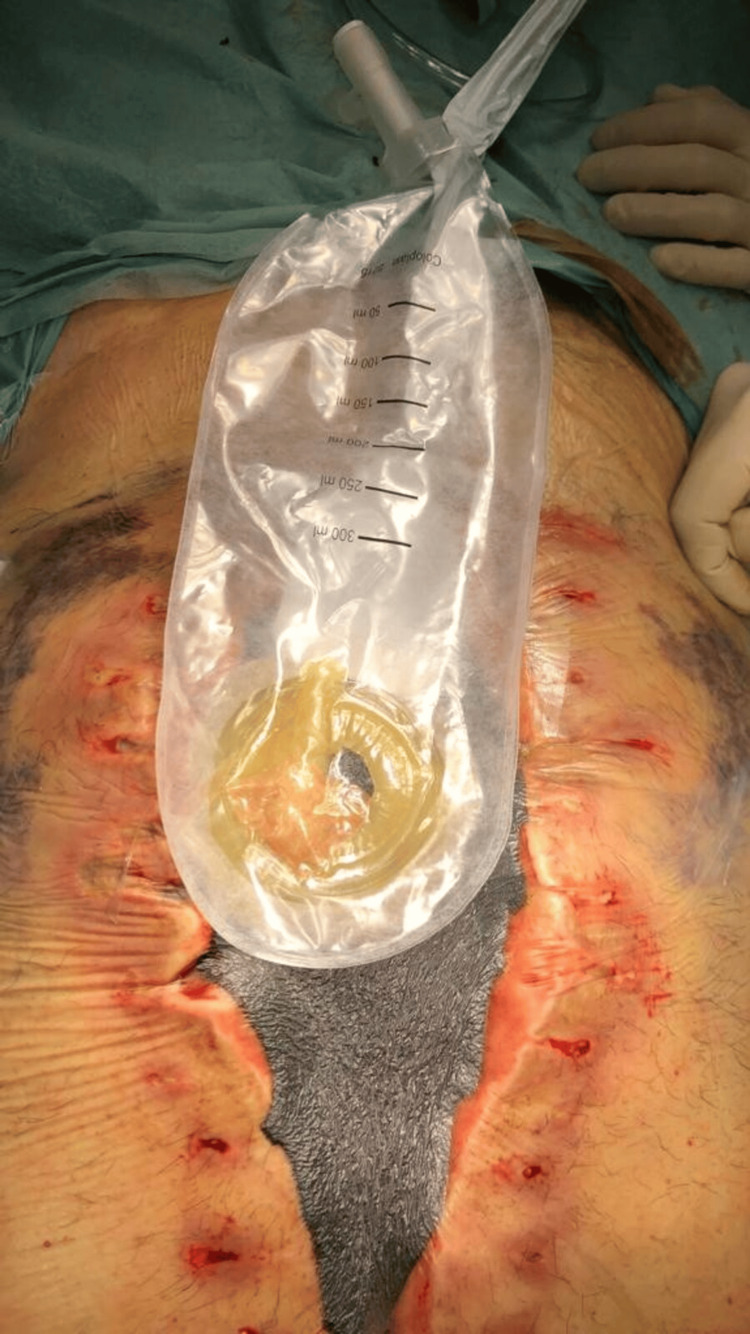
Ostomy bag application

In this final step, an ostomy bag is positioned beneath the protective ring, with the condom placed inside, to collect the enteric drainage (Figure 7).

Subsequent dressing changes were performed in the operating room, initially under general anesthesia and later under sedation and analgesia. The frequency of dressing changes was determined by issues with the dressing system, including vacuum system leaks, obstruction, and leakage at the EAF-condom anastomosis due to suture dehiscence. On average, the dressing was changed 1 to 2 times per week in this case.

**Figure 8:**
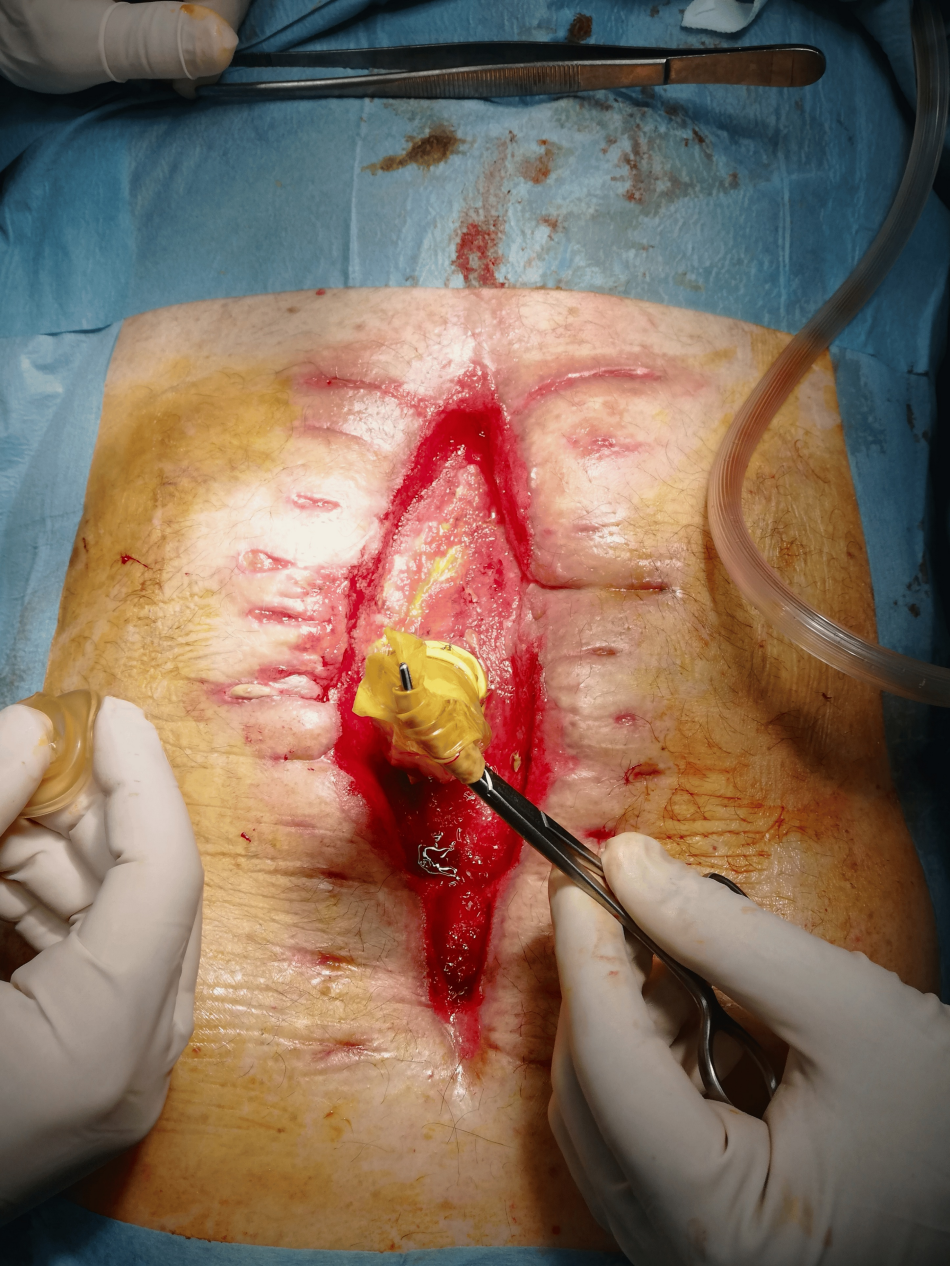
Secondary anastomosis between the enteroatmospheric fistula opening and a rubber drain to reduce leaks

Approximately midway through the treatment course for converting an EAF to an enterocutaneous fistula (ECF), we decided to modify the initial technique. Following the EAF-condom anastomosis, we performed a secondary anastomosis between the EAF opening and a rubber drain, to reinforce the anastomosis and reduce leaks. This adjustment allowed us to decrease the frequency of dressing changes to approximately once per week (Figure 8).

**Figure 9:**
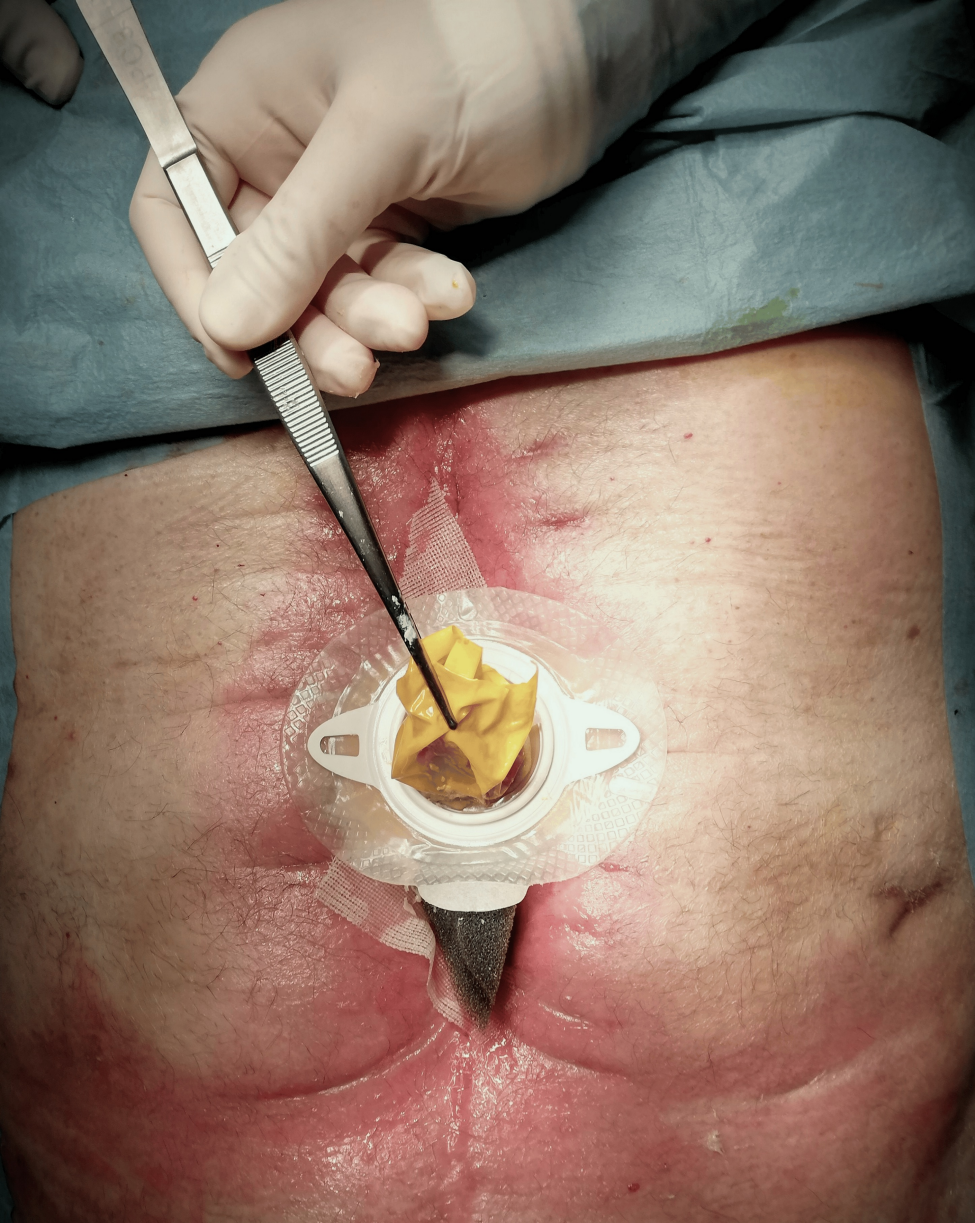
Ostomy plate applied directly around the condom

**Figure 10:**
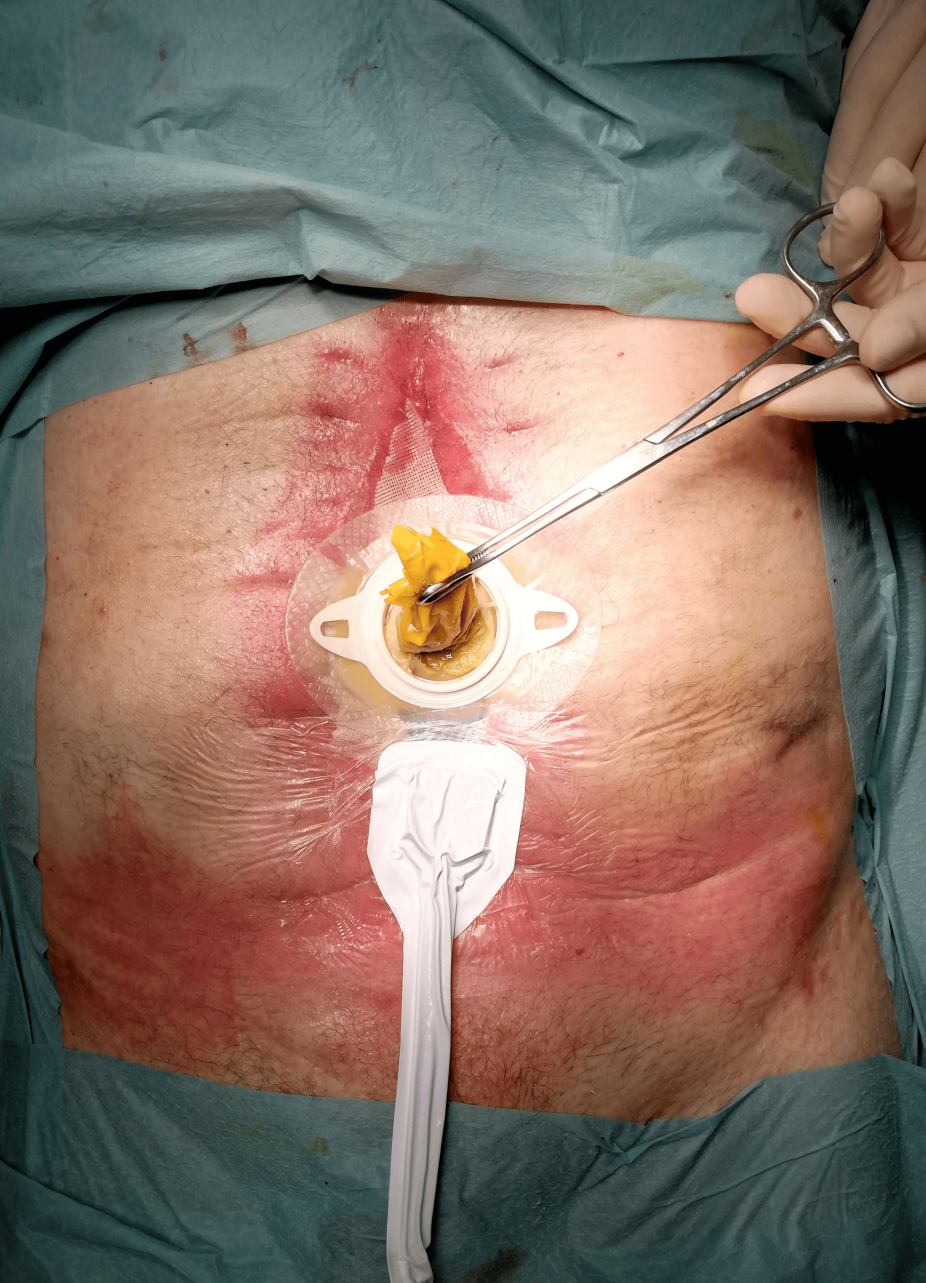
Negative pressure wound therapy foam applied in the remaining wound area

As the diameter of the wound decreased, new adaptations were necessary due to the reduced space. For instance, toward the final phase, we discontinued the use of the protective ring and instead applied an ostomy plate directly around the condom, while using NPWT foam to cover the remaining wound area (Figure 9, Figure 10). 

**Figure 11:**
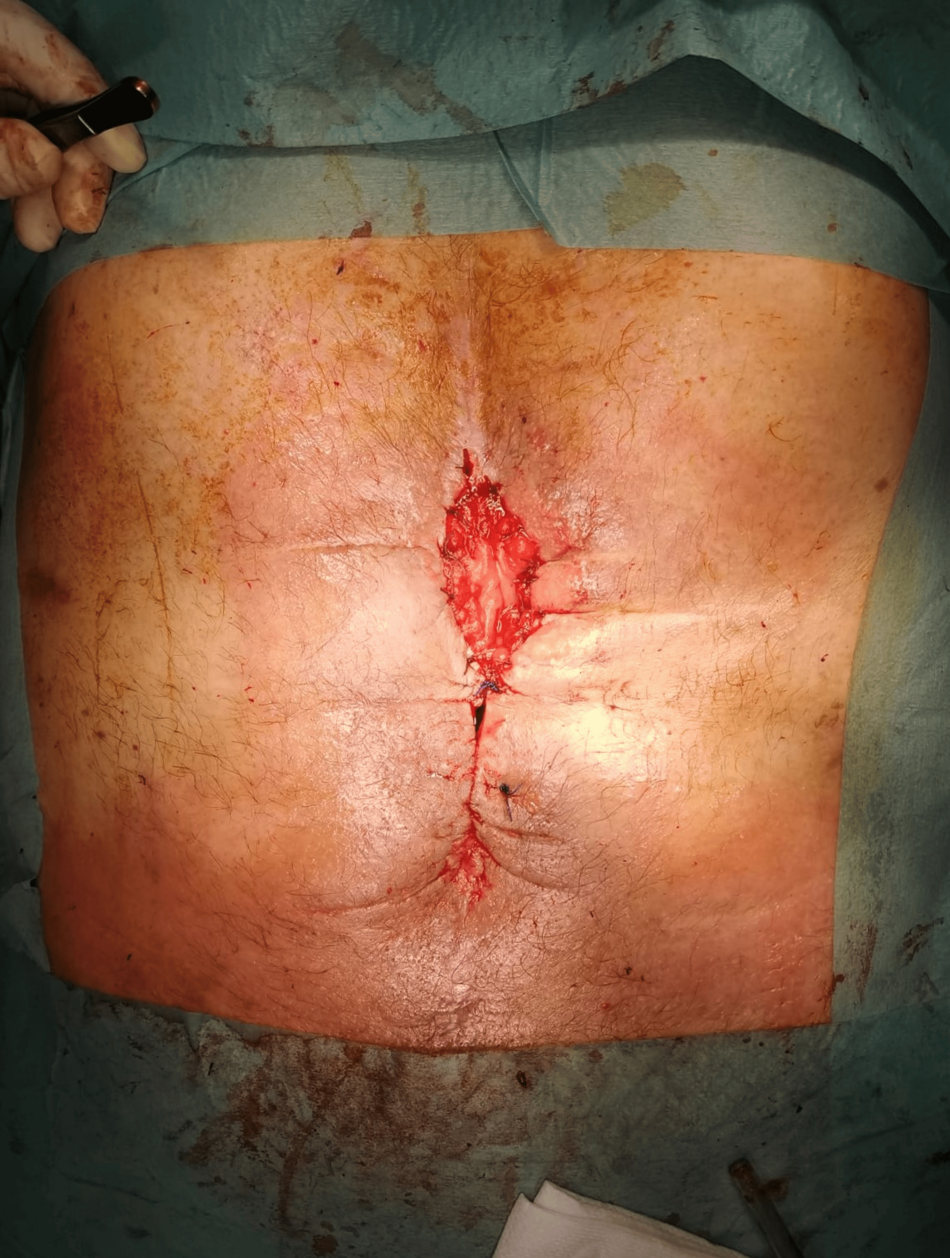
Enterocutaneous fistula (true stoma)

Approximately nine weeks after initiating therapy with the use of the condom technique, it was possible to convert the EAF to na ECF (true stoma). This involved the maturation of the ileostomy with absorbable 3-0 sutures and the approximation of the remaining wound edges with additional non-absorbable suturing (Figure 11).

Concurrently with the treatment, the patient received nutritional support and a nephrology consultation for the correction of the acute kidney injury and electrolyte disturbances associated with his high-output fistula. In addition, octreotide was administered to reduce the output. Ostomy assistance was also provided during jejunostomy maturation. 

Approximately one year after the initial surgery, and following optimization of the patient’s physical, nutritional, and metabolic status, the patient underwent a midline laparotomy with resection of the stoma area (around 15 cm). A primary mechanical enteric anastomosis was performed, latero-lateral, and isoperistaltic, thereby restoring intestinal continuity.

## Discussion

The variety of treatment options for EAF highlights the absence of consistent data, evidence-based outcomes, and a standardized approach. Several non-surgical techniques have been described. Endoscopic clipping has shown some success, with the largest series reporting a closure rate of 42.9% [11]. Fistula plugs, typically made from porcine mucosa and placed endoscopically, can achieve closure in up to 80% of the cases [12]. In addition, fibrin sealants have been documented in the literature for closing fistulas [13].

When opting for a surgical technique, it is crucial to focus on isolating the EAF opening and preventing contamination of the wound by fistula effluent.

NPWT has gained significant prominence in treating this condition, with several series demonstrating its safety. However, some case reports suggest that NPWT might lead to new fistula formation [14,15], with rates potentially reaching 14.6% [8].

Several surgical options have been described for EAF management. The floating stoma technique, first described by Subramaniam et al. in 2002 [16], has been used but was not applicable in this case due to the difficulty of exteriorizing the small intestine loop through the abdominal wall without tension.

The tube-NPWT technique involves inserting a drainage tube directly into the fistula opening and threading it through the NPWT foam to collect effluent. However, this method carries the risk of fistula enlargement and is not suitable for fistulas larger than the catheter [17].

The baby bottle nipple NPWT technique uses a modified nipple, typically made from silicone or rubber, to create a controlled environment for NPWT around the fistula [18]. We found it challenging to isolate the effluent from the rest of the abdominal wound.

Devices specifically designed to treat EAFs have been developed including the silicone fistula adapter (invented by PPM Fistelapater) and various fistula stents [19]. Those devices were not available at our hospital.

The technique described in this case report addresses the key challenges associated with EAFs. The first step diverts the effluent away from the abdominal wound, protecting it from the corrosive effects of enteric or pancreatic contents. The second step involves creating a protective ring to prevent negative pressure from affecting the condom and fistula, allowing passive drainage into a collection bag. In the third step, NPWT is applied to enhance granulation, wound contraction, exudate removal, and control the microenvironment, thereby accelerating healing by secondary intention [20]. Finally, the fourth step focuses on managing the fistula's contents.

This technique has limitations, including multiple operating room visits due to frequent vacuum system leaks or obstructions. There is also an inherent risk of laceration to the fistula’s edges due to frequent suturing. Despite these drawbacks, it remains a relatively easy-to-reproduce technique that uses materials commonly available in hospitals. It can be implemented even in facilities with limited resources while being safe and relatively inexpensive.

## Conclusions

The described four-step technique offers a practical and reproducible approach to managing EAFs, effectively addressing the core challenges of effluent control, wound protection, and prevention of contamination. This method enables safer wound management by directing effluent away from the abdominal wound while promoting a favorable environment for healing. The use of readily available materials makes this approach accessible, even for hospitals with limited resources, supporting its potential for broader clinical application.

While the technique has inherent limitations, it remains a viable solution for treating complex EAFs in critical settings. Its adaptability and simplicity offer a valuable addition to the available treatment strategies, presenting a cost-effective and efficient alternative that aligns with the multidisciplinary efforts required in EAF management.
